# Hospitalized Patients at High Risk for Obstructive Sleep Apnea Have More Rapid Response System Events and Intervention Is Associated with Reduced Events

**DOI:** 10.1371/journal.pone.0153790

**Published:** 2016-05-11

**Authors:** Sunil Sharma, Anindita Chowdhury, Lili Tang, Leslee Willes, Brian Glynn, Stuart F. Quan

**Affiliations:** 1 Thomas Jefferson University, Philadelphia, Pennsylvania, United States of America; 2 Harvard Medical School, Boston, Massachusetts, United States of America; 3 University of Arizona College of Medicine, Tucson, Arizona, United States of America; 4 Willes Consulting Group Inc., Encinitas, California, United States of America; National Institute for Viral Disease Control and Prevention, CDC, China, CHINA

## Abstract

**Background:**

Rapid response system (RRS) is a safety tool designed for early detection and intervention of a deteriorating patient on the general floor in the hospital. Obstructive sleep apnea (OSA) has been associated with significant cardiovascular complications. We hypothesized that patients with high-risk of OSA have higher rate of RRS events and intervention with positive airway pressure therapy in these patients can mitigate the RRS events.

**Methods:**

As part of a clinical pathway, during a 15 month period, patients with BMI ≥ 30 kg/m^2^ in select medical services were screened with a validated sleep questionnaire. Patients were characterized as high or low risk based on the screening questionnaire. RRS rates were compared between the groups. Subsequently the impact of PAP therapy on RRS events was evaluated.

**Results:**

Out of the 2,590 patients screened, 1,973 (76%) were identified as high-risk. RRS rates calculated per 1,000 admissions, were 43.60 in the High-Risk OSA group versus 25.91 in the Low-Risk OSA Group. The PAP therapy compliant group had significantly reduced RRS event rates compared to non-compliant group and group with no PAP therapy (16.99 vs. 53.40 vs. 56.21) (p < 0.01).

**Conclusion:**

In a large cohort of patients at a tertiary care hospital, we show an association of increased rate of RRS events in high-risk OSA patients and reduction of the risk with PAP intervention in the compliant group.

## Introduction

Rapid response systems (RRS) are commonly used safety protocols in hospitals throughout the United States. These systems are designed to identify and quickly intervene to reverse serious and potentially fatal deterioration of patients in the general hospital population. Data reveal that RRS are associated with reduction in rates of cardiopulmonary arrests [1]

Obstructive sleep apnea (OSA) is being increasingly recognized as a risk factor for cardiovascular complications and sudden death [2, 3]. There is a strong association of OSA with elevated body mass index (BMI) [4]. Our recent data shows that obese hospitalized patients have a high prevalence of undetected OSA [5]. We found that in obese hospitalized patients who were determined to be high risk for OSA on screening, 87% were found to have OSA on polysomnography performed post discharge. These findings are consistent with prior literature by Collop et al showing that 77% of patients referred from the hospital diagnostic testing had OSA.

Although there is scant literature on acute complications of OSA in hospitalized patients, recent data suggest that among patients admitted with pneumonia, OSA was associated with higher rates of mechanical ventilation and clinical deterioration [6]. Conversely, others found no clinical deterioration and reduced inpatient mortality. However these studies were done using billing codes to identify cases, and may have missed significant undiagnosed OSA [7, 8].

We hypothesized that patients with high risk of OSA have a greater likelihood of acute complications as measured by RRS events. The purpose of this study was to determine whether hospitalized patients at high risk for OSA have a greater rate of RRS events, and whether intervention with positive airway pressure devices was associated with reduction of these events.

## Methods

During the period from April 2013 through Jan 2015, as part of a clinical pathway, all obese (BMI ≥ 30 kg/m^2^) patients admitted to Thomas Jefferson University Hospital Monday through Friday on three targeted services (Internal Medicine/ Family practice/ Cardiology [Specialty Group]) were screened for OSA by the STOP questionnaire (Snoring, Tiredness, Observed apneas and Blood Pressure)[9]. This instrument consists of 4 brief items documenting the presence or absence of snoring, daytime tiredness (including fatigue and sleepiness), witnessed apnea and hypertension. It has been validated in high risk and general populations and found to have good sensitivity, specificity and a high predictive value for the identification of OSA [10]. The questionnaire was administered, scored and documented in the electronic medical record (EMR) by hospital respiratory therapists. Those patients who were screened positive (Yes to 2 or more questions on the 4 question instrument) were labeled high risk and the admitting team / providers notified. The admitting team/ providers then had the option, but were not required, to obtain a Pulmonary/Sleep Medicine consultation. If consultation was requested, a comprehensive sleep evaluation was conducted and included inpatient management with non-invasive positive pressure ventilation, as required. Patients admitted to the hospital on Saturday, Sunday or holidays or who were not obese (<30 kg/m^2^) did not undergo screening. Patients with known diagnosis of OSA and on positive airway pressure (PAP) therapy were excluded from this analysis. PAP refers to continuous positive airway pressure (CPAP), auto-adjusting positive airway pressure (APAP) or bilevel positive airway pressure (bilevel PAP). This retrospective analysis study was reviewed and approved by the Thomas Jefferson University institutional review board. Patient consent was not obtained as the data collected from electronic medical records was anonymized and de-identified prior to analysis.

During the evaluation period, data from RRS records of the screened patients was extracted to determine the rate of RRS events in following the categories: 1) high risk OSA as classified by the STOP questionnaire; 2) low risk OSA as classified by the STOP questionnaire. The adult RRS events at Thomas Jefferson University Hospital are defined as 1) significant blood pressure changes from baseline (<90 or >180 mm Hg systolic pressure); 2) significant heart rate change from a patient’s baseline (<40 or >130 beats per minute); 3) significant respiratory rate change (<8 or >24 breaths / minute); 4) unexpected oxygen desaturation < 88%; 5) urine output <50 cc in 4 hours; 6) mental status change, seizure or symptoms of stroke; 7) chest pain with hypo or hypertension; or 8) staff member concern. In the high risk OSA category, patients were further classified as receiving or not receiving PAP for treatment of OSA. For the PAP treated patients, usage was assessed by examining the daily respiratory therapist note, in the EMR. Patients were considered compliant with treatment if the respiratory therapist note reported usage of PAP for 4 hours or more during sleep. Compliance was ascertained through interviews by the respiratory therapist with the night staff nurse and patient. In addition to RRS event rate, length of stay (LOS) for patients in these groups was determined through review of the EMR.

## Statistical Analysis

Patients were assigned to two groups: High Risk OSA and Low Risk OSA. Pairwise RRS rates per 1000 admissions were then compared among the High Risk OSA Group and the Low Risk OSA Group using a Poisson rates model. Within the High Risk OSA Group, RRS rates were compared among patient groups who were PAP compliant, PAP non-compliant and those who declined PAP therapy also using a Poisson rate model.

The mean LOS was compared in the High Risk OSA Group and the Low Risk OSA Group with analysis of variance. The LOS also is presented for the overall hospital for reference purposes.

Inasmuch as it was not logistically possible to obtain individual level demographic, anthropometric and comorbidity data on all patients admitted to the Specialty Group during the evaluation period for the study, a representative subsample of patients was selected to assess whether there were differences in these characteristics between patients in the High Risk OSA group and patients who were in the Low Risk OSA group. A sample size of 64 patients in each group was selected to yield 80% power to detect a medium effect size for these characteristics using comparisons of means or proportions with a type I error rate of 0.05 [11]. The 64 patients in the High Risk OSA group were equally stratified into those patients having a sleep consultation (n = 32) and patients not having a sleep consultation (n = 32). Baseline characteristics were compared using a two-sided t-test for continuous parameters and a chi-square or Fisher’s exact test for categorical parameters. Data are shown as mean ± SD. P<0.05 was considered statistically significant.

## Results

**Fig 1:**
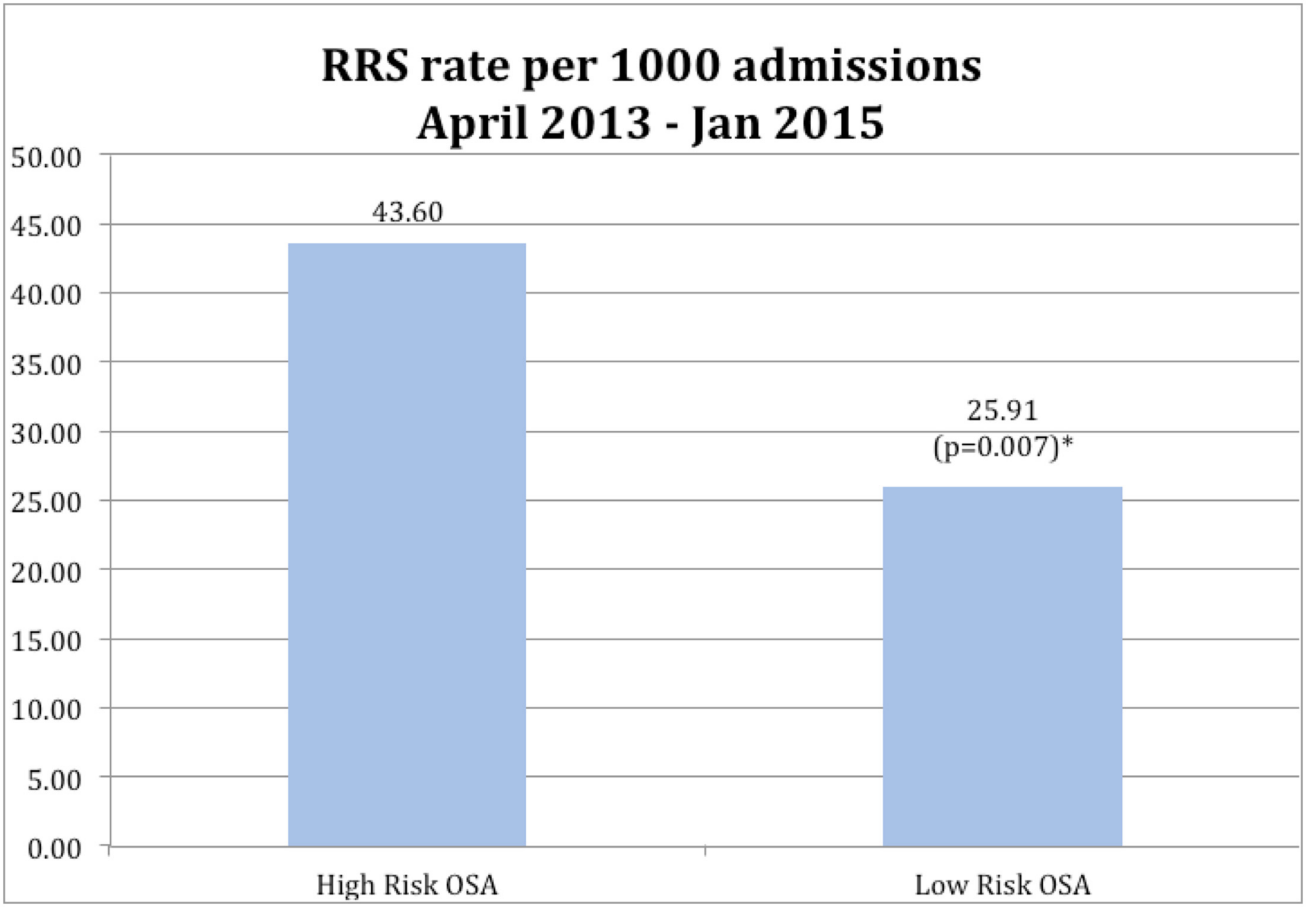
Rapid response system (RRS) events in High Risk OSA patients versus Low Risk OSA patients.

A total of 2,590 patients were screened during the period between April 2013 through Jan 2015 with the STOP questionnaire. Out of 2,590, 1,973 patients were identified as high risk for OSA. In this High Risk OSA Group, there were 157 RRS events compared to 32 in the Low Risk OSA Group. Because some patients were admitted on multiple occasions during the evaluation period, the total number of admissions in these groups were as follows, High-Risk OSA Group: 3,601, Low Risk OSA Group: 1,235;RRS rates calculated per 1000 admissions were 43.60 in the High-Risk OSA group versus 25.91 in the Low Risk OSA Group (all paired comparisons between High Risk OSA and low risk OSA were statistically significant, p = 0.007) (Fig 1). The main reason (69%) for RRS events in the high-risk population were cardio-pulmonary (arrhythmia, hypoxia, hypotension, syncope, respiratory distress), while in the low-risk population 50% of the RRS events were cardio-pulmonary.

**Fig 2:**
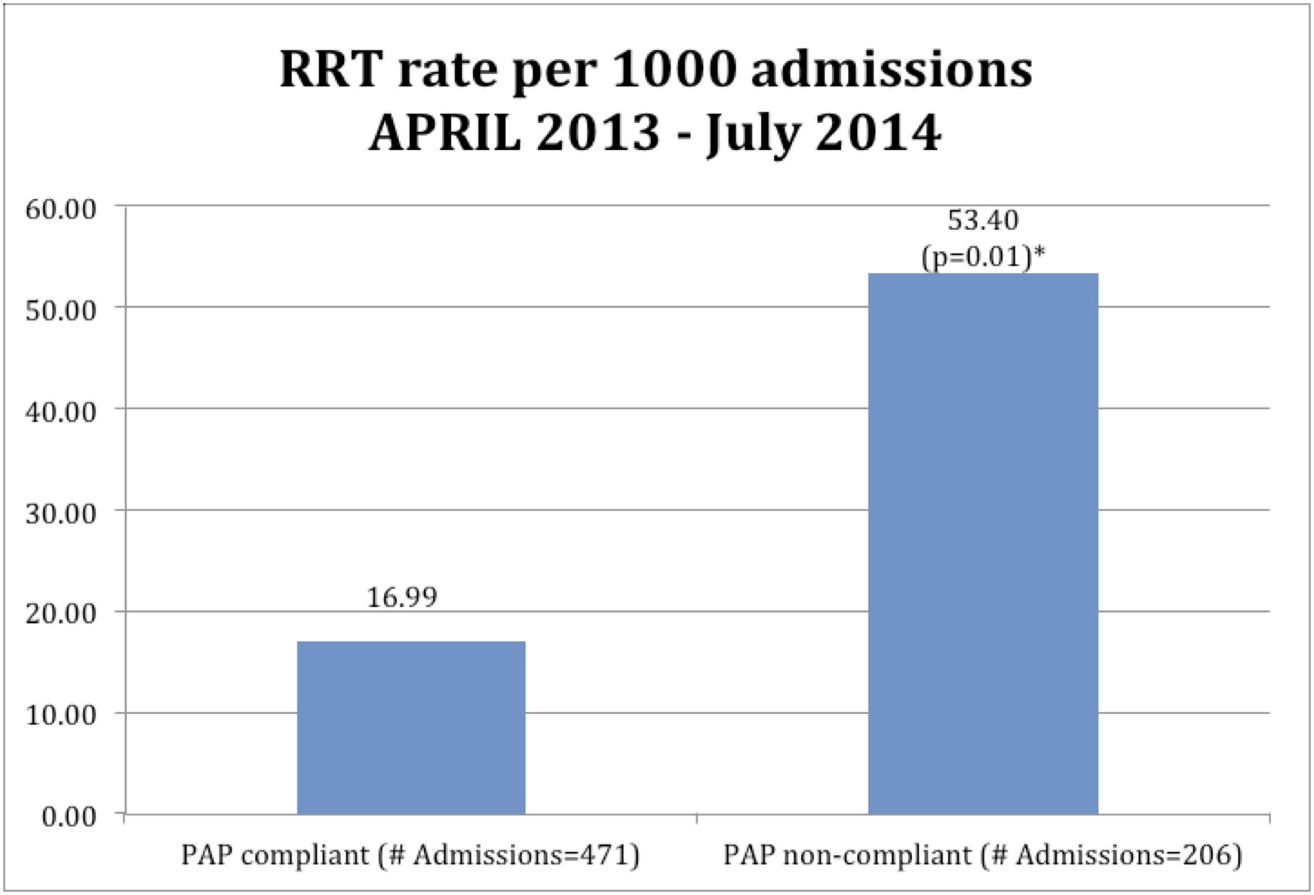
Comparison between patients who were compliant with PAP therapy versus non-compliant, from April 2013 to July 2014.

Among the 1,973 High-Risk OSA group, 919 admissions received in-hospital consultation by the Pulmonary Sleep Medicine service. Of the 919 admissions, 677 admissions were recommended and received PAP therapy. Of these 677 admissions who received PAP therapy, 471 were compliant with therapy and 206 were non-compliant. The decision to recommend / initiate PAP therapy was based on results of overnight pulse-oximetry and clinical risk assessment. As shown in Fig 2, those who were compliant with PAP therapy had significantly reduced RRS event rates compared to the group with non-compliance or the group with no PAP therapy (16.99 vs 53.40 vs 56.21) (PAP Compliant vs No PAP, PAP Compliant vs PAP Non-compliant, p < 0.01).

Of the 207 (73%) patients who were compliant on PAP, 112 (54%) were on fixed CPAP, 35 (17%) on APAP and 60 (29%) on Bilevel PAP. The patients were initiated on CPAP in majority of the cases, but BiPAP was instituted for either difficulty in tolerating CPAP or need to treat concomitant hypercapnea. Similarly Auto-PAP was used if CPAP was not tolerated and only OSA was suspected.

**Fig 3:**
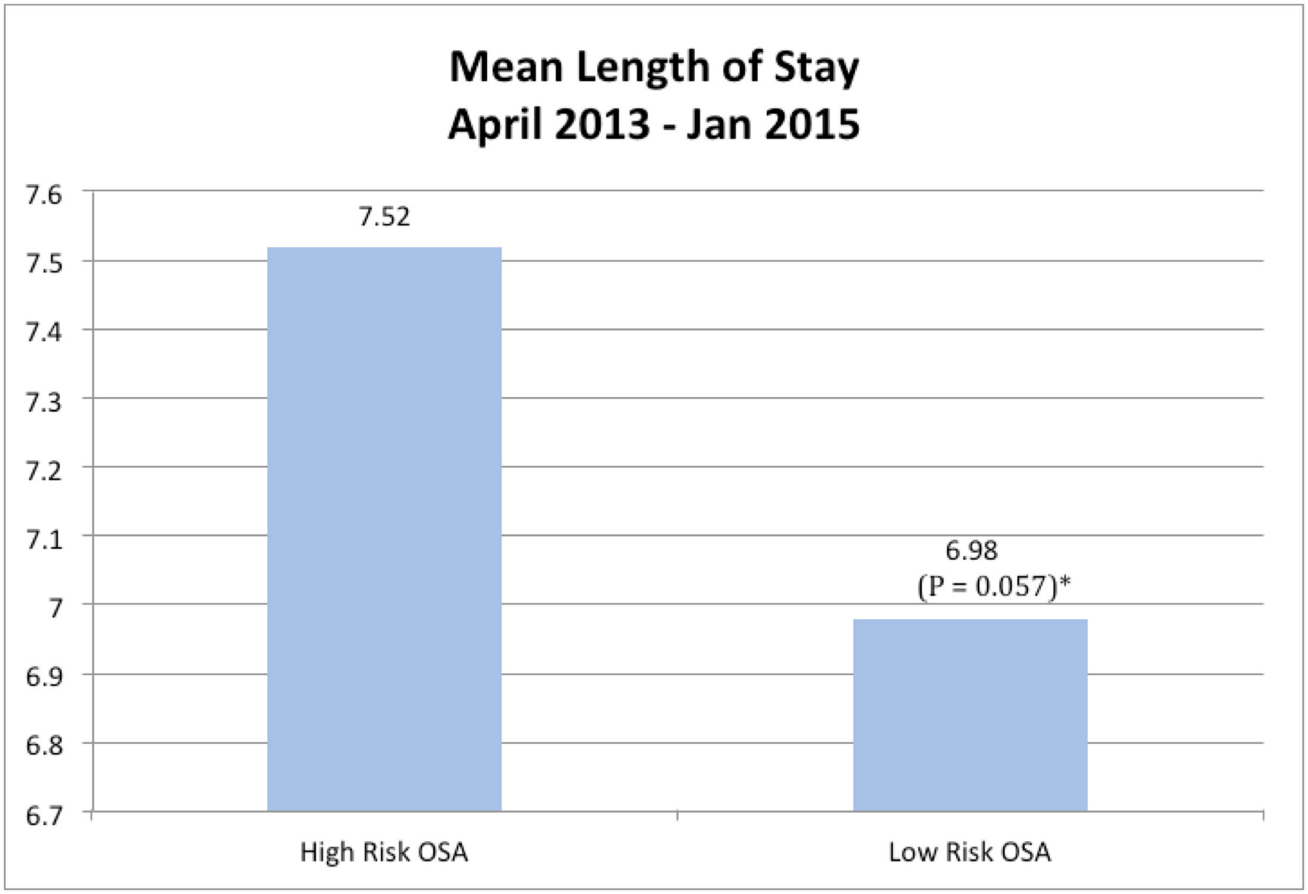
Comparison of mean length of stay in patients who were high risk for OSA versus low risk for OSA.

The mean LOS in the High-Risk OSA group was determined to be significantly higher than the mean LOS in the Low-Risk OSA group. (Fig 3)

In Table 1 shows the demographic and clinical characteristics of a subset of High Risk OSA and Low Risk OSA patients. High-risk OSA patients were more likely to be African American, male and had evidence for hypertension, congestive heart failure and coronary artery disease. High-risk patients had higher mean BMIs than low-risk patients but this difference was not significant. However, the prevalence rates of type 2 diabetes mellitus and chronic obstructive lung disease were similar between groups.

**Table 1:** Baseline characteristics comparison between sample of High-Risk and Low-Risk OSA patients selected randomly.

	Mean±SD, Median / n(%)		
Characteristic	STOP Negative (N = 64)	STOP Positive (N = 64)	p-value
Age (years)	61±18, 61	61±14, 61	0.92
Body mass index (BMI)	34±7, 33	37±9, 35	0.09
Male	20 (31)	32 (50)	0.03
Black	18 (30)	40 (63)	0.0002
Coronary artery disease (CAD)	11 (17)	20 (31)	0.06
Chronic kidney disease (CKD)	11 (17)	19 (30)	0.10
Hypertension (HTN)	32 (50)	51 (80)	0.0004
Congestive Heart Failure (CHF)	16 (25)	37 (58)	0.0002
Chronic Obstructive Pulmonary Disease (COPD)	4 (6)	8 (13)	0.36
Diabetes Mellitus Type 2 (DM)	25 (39)	32 (50)	0.21
Pulmonary Hypertension (PH)	5 (8)	3 (5)	0.72
Beta- blockers	29 (45)	36 (56)	0.22
Angiotension-Converting-Enzyme Inhibitor (ACE-I)	17 (27)	34 (53)	0.002
Calcium Channel Blocker (CCB)	12 (19)	16 (25)	0.39
Diuretics	23 (36)	36 (56)	0.02

## Discussion

To our knowledge, this is the first study to find a significantly greater rate of RRS events in hospitalized patients who are high-risk for OSA in comparison to those at lower risk. Furthermore, our study suggests that early intervention with PAP in this group can reduce RRS events.

RRS intervention is increasing being used across hospitals in the United States as part of quality improvement initiatives [12]. The RRS team can consist of medical personal from different disciplines and can intervene emergently and independent of the admitting providers [13]. Recent studies have revealed that use of a RRS is associated with reduction in cardiopulmonary arrests in non-ICU adult patients [1].

Patients with OSA are at increased risk for cardiovascular complications including sudden death, fatal and non-fatal arrhythmias and acute coronary syndrome [3,14]. This may be true particularly in a hospital setting where it has been demonstrated that OSA patients have higher postoperative complications [15]. Using the STOP questionnaire in our prior study, we found a high burden of OSA in obese hospitalized patients potentially placing these patients at greater risk for unanticipated adverse outcomes while hospitalized [5]. This possibility was confirmed by our finding in this analysis that these patients had a greater likelihood of having RRS events. STOP questionnaire was used because of its simplicity, ability to self- administer and our Institutions experience using the questionnaire [16].

Another major finding of our study was that PAP therapy was associated with a reduced frequency of RRS events in compliant patients. In ambulatory cohorts, intervention with CPAP decreases both cardiovascular-related and overall mortality [17, 18, 19]. In postoperative OSA patients, the putative benefits of CPAP intervention are unsettled [20]. Moreover, whether CPAP intervention is effective in reducing hospital mortality in previously untreated and/or undiagnosed patients is not known. Nevertheless, our results are consistent with a recent report demonstrating that bariatric surgery patients who had a positive STOP-BANG screen for OSA and were not treated with CPAP had a higher rate of pulmonary complications and greater LOS than patients with a polysomnography confirmed diagnosis of OSA and who were treated with CPAP [21]. This observation, in conjunction with our findings, suggests that aggressive identification and treatment of hospitalized patients at high risk for OSA can decrease adverse outcomes and thus reduce hospital costs.

We observed that LOS was greater in those at higher risk for OSA. Given that high risk for OSA likely is a marker of illness severity, a greater LOS is not surprising. Whether CPAP intervention can reduce the LOS has so far been unclear. Previous studies using large databases suggest that LOS in patients using CPAP is higher and indicative of more complex disease [22]. However, more recent findings suggest that use in postoperative patients may lead to a decrease in LOS [20]. Our data support the latter observation.

Not surprisingly, our analysis of a subset of High Risk OSA and Low Risk OSA patients found those with High Risk OSA were more likely to have heart disease, congestive heart failure and hypertension. Thus, High Risk for OSA could merely be a surrogate for other acute and chronic disease processes that increase the probability of a RRS event. We do not believe this is the entire explanation for our findings because in High Risk OSA patients who received PAP therapy, the likelihood of a RRS event was much lower.

Our study has several limitations. First, it is an observational study. However, the study had a large cohort of patients and used a well-defined clinical pathway. Second, the screening program was conducted only during weekdays. However we have no reason to believe that the patient demographics and profile at our institution are significantly different during the weekends. Third, the compliance of the patients with PAP therapy was assessed by the observations of the respiratory therapists and not by objective data download. However, compliance was determined in the morning after interviewing the patient and the overnight nurse by trained respiratory therapists and documented in the EMR, and thus was subject to minimal recall bias. Lastly, because it was not possible to obtain individual level demographic, anthropometric and comorbidity data, our results were not adjusted for baseline characteristics, severity of illness or reasons for admissions. However, we believe our sub analysis of these factors accurately describes our cohort, and the lower RRS event rate in the PAP compliant group suggests that differences in baseline factors do not entirely explain our findings. Despite these limitations, strength of our study is the prospective screening of patients instead of the reliance on ICD-9 codes. Nevertheless, because the STOP instrument does not have 100% sensitivity and specificity, some misclassification error might have occurred, although it was likely nondifferential.

## Conclusions

In a large cohort of patients at a tertiary care hospital, we showed that there was an increased rate of RRS events in high-risk OSA patients and that PAP intervention was associated with decreased frequency of RRS events. The STOP questionnaire is a simple screening tool for OSA and can be implemented by hospitals with minimal financial burden and expertise [9] Thus, implementation of a high-risk OSA screening program in hospitals should be cost effective. Further prospective, multi-centric studies are advised to confirm these findings.
